# Temperature responsiveness of gilthead sea bream bone; an *in vitro* and *in vivo* approach

**DOI:** 10.1038/s41598-018-29570-9

**Published:** 2018-07-25

**Authors:** Natàlia Riera-Heredia, Rute Martins, Ana Patrícia Mateus, Rita A. Costa, Enric Gisbert, Isabel Navarro, Joaquim Gutiérrez, Deborah M. Power, Encarnación Capilla

**Affiliations:** 1 Departament de Biologia Cel·lular, Fisiologia i Immunologia, Facultat de Biologia, Universitat de Barcelona, 08028 Barcelona, Spain; 2 Centro de Ciências do Mar (CCMAR), Universidade do Algarve, Campus de Gambelas, 8005-139 Faro, Portugal; 3 Institut de Recerca i Tecnologia Agroalimentàries (IRTA), 43540 Sant Carles de la Ràpita, Spain

## Abstract

This study aimed to characterize the molecules involved in osteogenesis in seabream and establish using *in vitro*/*in vivo* approaches the responsiveness of selected key genes to temperature. The impact of a temperature drop from 23 to 13 °C was evaluated in juvenile fish thermally imprinted during embryogenesis. Both, *in vitro*/*in vivo*, *Fib1a*, appeared important in the first stages of bone formation, and *Col1A1*, *ON* and *OP*, in regulating matrix production and mineralization. *OCN* mRNA levels were up-regulated in the final larval stages when mineralization was more intense. Moreover, temperature-dependent differential gene expression was observed, with lower transcript levels in the larvae at 18 °C relative to those at 22 °C, suggesting bone formation was enhanced in the latter group. Results revealed that thermal imprinting affected the long-term regulation of osteogenesis. Specifically, juveniles under the low and low-to-high-temperature regimes had reduced levels of *OCN* when challenged, indicative of impaired bone development. In contrast, gene expression in fish from the high and high-to-low-temperature treatments was unchanged, suggesting imprinting may have a protective effect. Overall, the present study revealed that thermal imprinting modulates bone development in seabream larvae, and demonstrated the utility of the *in vitro* MSC culture as a reliable tool to investigate fish osteogenesis.

## Introduction

Bone formation in fish does not occur during somitogenesis and the first skeletal structures appear after hatching. These structures consist of dermal bone (formed by intramembranous ossification) and cartilage replacement bone. The skeleton of the jaw and mouth only form after mouth opening with the onset of exogenous feeding. Subsequently, the first vertebrae appear in larvae at around 4 mm in body length, and only after early flexion (6 mm) does mineralization of these structures start to take place^1–4^. An almost fully mineralized skeleton is evident in larvae of 16 mm body length^1^ or at about 30 days post-hatching^4^. Concerning the molecules involved in the formation and turnover of bone, transcriptomes from gilthead sea bream (*Sparus aurata*) gill arch and vertebra have recently been generated and revealed homologs of many mammalian skeleton-related transcripts^5^. Many of the identified genes corresponded to transcription factors that control osteoblast differentiation (i.e. runt-related transcription factor 2 (Runx2/Cbfa1), osterix/Sp7), or components of the extracellular matrix (ECM), including structural proteins, collagens, proteoglycans, as well as non-collagenous proteins that regulate mineral deposition such as osteonectin/SPARC (ON), osteopontin/Spp1 (OP) and osteocalcin/BGP (OCN). The study by Vieira *et al*.^5^, unveiled the conservation of transcripts present in bone and cartilage of gilthead sea bream compared to mammals, although the tissue and cell specific localization of these transcripts remains to be established. However, with the increasing number of *in vitro* fish cell models developed in recent years^6,7^, including the gilthead sea bream primary culture of mesenchymal stem cells (MSCs) derived from vertebrae, established by our group^8^, it is now possible to characterize at a cellular level the role of identified candidate markers during osteoblastogenesis.

Gilthead sea bream is one of the most important farmed species in Spain, which is ranked fourth in total production volume in the Mediterranean area and third in the European Union^9^. Although the production of gilthead sea bream in aquaculture systems is well consolidated, the high incidence of skeletal deformities is still an important bottleneck for the sustainability of this industry. Moreover, since this species is commercialized as whole fish, skeletal anomalies lead to a downgrade in the quality of the product and a reduction in its value. The most common deformities found in gilthead sea bream are those that affect the opercular complex, vertebral column and haemal or caudal body regions^10–12^, which may result in ca. to 50% of lost production at the end of the hatchery phase^13^. These deformities may also reduce growth rate, increase mortality and negatively impact animal welfare^14^.

The aetiology of skeletal deformities is uncertain, but temperature is one of the most important abiotic factors linked to this problem and fish grown at elevated temperatures exhibit increased levels of vertebral anomalies^13–15^. In fact, fluctuations in water temperature are associated with abnormal muscle growth and heterochrony of muscle formation, whereas temperature effects on bone growth have been associated with deformation of the vertebral bodies in fish larvae, which leads to spinal deformities^16,17^. In addition, body shape and meristic characters (i.e. dorsal spines) are also significantly affected by environmental temperature during the early life stages of the European sea bass, *Dicentrarchus labrax*^18^. In Atlantic salmon (*Salmo salar*), faster growth induced by hyperthermia significantly modified gene transcription in osteoblasts and chondrocytes, which was associated with an increased number of deformities and modified bone tissue structure and composition^19^.

In the Mediterranean Sea, gilthead sea bream is frequently exposed to severe temperature changes during winter, which is one of the causal factors of winter syndrome in reared populations^20^. This pathology causes chronic mortality during the coldest months of the year and acute death episodes when the water temperature rises again^21,22^. Mortality rates are usually around 7–10% of the fish stock, although in some very acute cases, they may be as high as 80%^23^. Winter syndrome is a multifactorial condition that triggers a stress response^24,25^, depresses the immune system^21,26^, affects several tissues including the skeletal muscle, exocrine pancreas, liver and digestive tract^20,25,27,28^, and may cause lesions in the brain and kidney^23^. The effect of thermal imprinting in embryos and larvae and on the response of the skeleton to a temperature drop (to simulate winter conditions) was recently studied in adult gilthead sea bream^29^. Overall, the study reported that although thermal imprinting failed to modify bone homeostasis in optimal ambient water temperatures, it did change the bones responsiveness during a cold challenge.

Following on from our previous work, in the present study, we addressed the hypothesis that, modifications in the skeleton due to water temperature are linked to its effect on the gene expression of key osteogenic molecules during development. To this end, we analysed *in vivo* how thermal regimes during development influenced the expression of genes involved in osteogenesis and used an *in vitro* primary culture of MSCs from juvenile gilthead sea bream vertebrae to investigate how temperature modified bone tissue specific gene expression. We then assessed *in vivo* how thermal regimes experienced during early development influenced the expression of bone tissue specific genes in adults exposed to a cold challenge.

## Results

### Bone cells development and temperature effects *in vitro*

**Figure 1:**
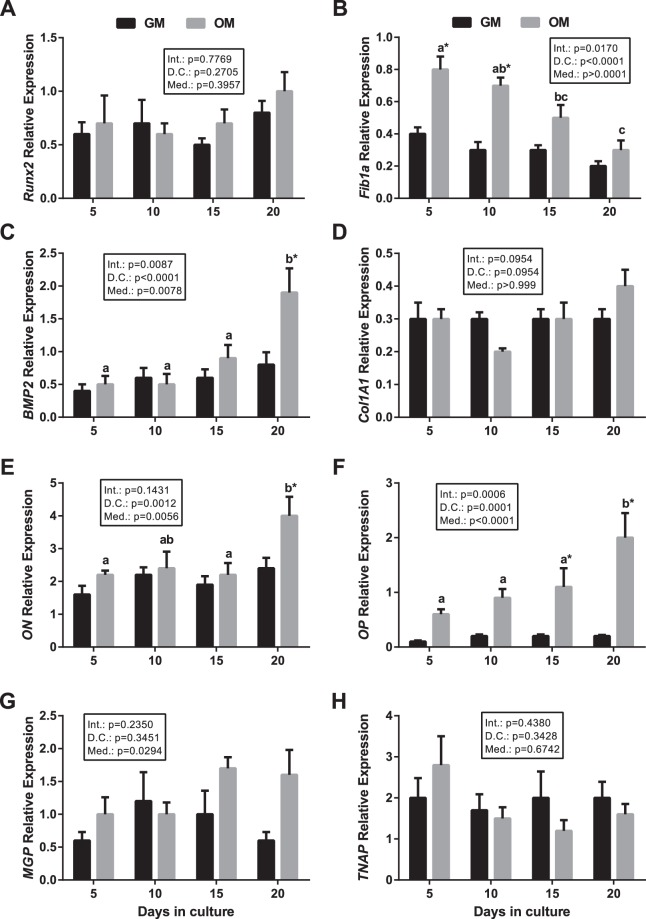
Expression of osteogenic genes in gilthead sea bream differentiating bone-derived primary cell cultures *in vitro*. Quantitative gene expression relative to the geometric mean of *RPS18* and *EF1α* for (**A**) *Runx2*, (**B**) *Fib1a*, (**C**) *BMP2*, (**D**) *Col1A1*, (**E**) *ON*, (**F**) *OP*, (**G**) *MGP* and (**H**) *TNAP* in cells cultured in growth (GM) or osteogenic (OM) media at days 5, 10, 15 and 20. The results are shown as the mean ± s.e.m. (n = 5–8). Different letters indicate significant differences throughout time within groups (upper case for GM and lower case for OM) and asterisks indicate significant differences between groups at each culture day (p < 0.05). Int.: Interaction, D.C.: Days in culture, Med.: Media.

Orthologs of mammalian osteogenic genes have recently been identified in gilthead sea bream by transcriptome analysis, however their specific expression in bone cells during the process of osteoblastogenesis remains elusive. The transcriptional profile of gilthead sea bream bone-derived MSCs differentiating into osteoblasts under mineralizing conditions induced by incubation with an osteogenic medium (OM) was compared to that of cells growing in growth medium (GM) for 20 days and is presented in Fig. 1. A significant interaction between days in culture and media was observed for fibronectin 1a (*Fib1a*), Bone Morphogenetic Protein 2 (*BMP2*) and *OP* mRNA levels. Nevertheless, significant differences were not observed with regard to transcript abundance through time in cells growing in GM for any of the genes studied (Fig. 1). Moreover, in cells incubated in OM, the gene expression of *Runx2*, a key transcription factor driving osteogenesis of MSCs, and of collagen type 1 alpha-1 (*Col1A1*), and tissue non-specific alkaline phosphatase (*TNAP*), did not vary significantly as differentiation progressed (Fig. 1A,D,H). Matrix Gla protein (*MGP*) showed similar results, but probably as an effect of the culture media (P = 0.029), an increase in its expression was observed at day 20 in cells growing in OM (Fig. 1G). *Fib1a* expression decreased gradually during MSCs differentiation, and significantly higher mRNA levels were found in the OM group compared to the GM group at days 5 and 10 (Fig. 1B). In contrast, *BMP2*, *ON* and *OP* expression increased during culture under mineralizing conditions and significantly higher mRNA levels were detected at day 20 relative to the previous days (Fig. 1C,E,F). A significant increase in transcript abundance in the OM group compared to the GM group was noted for *OP* at days 15 and 20 (Fig. 1F), and for *BMP2* and *ON* at day 20 (Fig. 1C,E).

**Figure 2:**
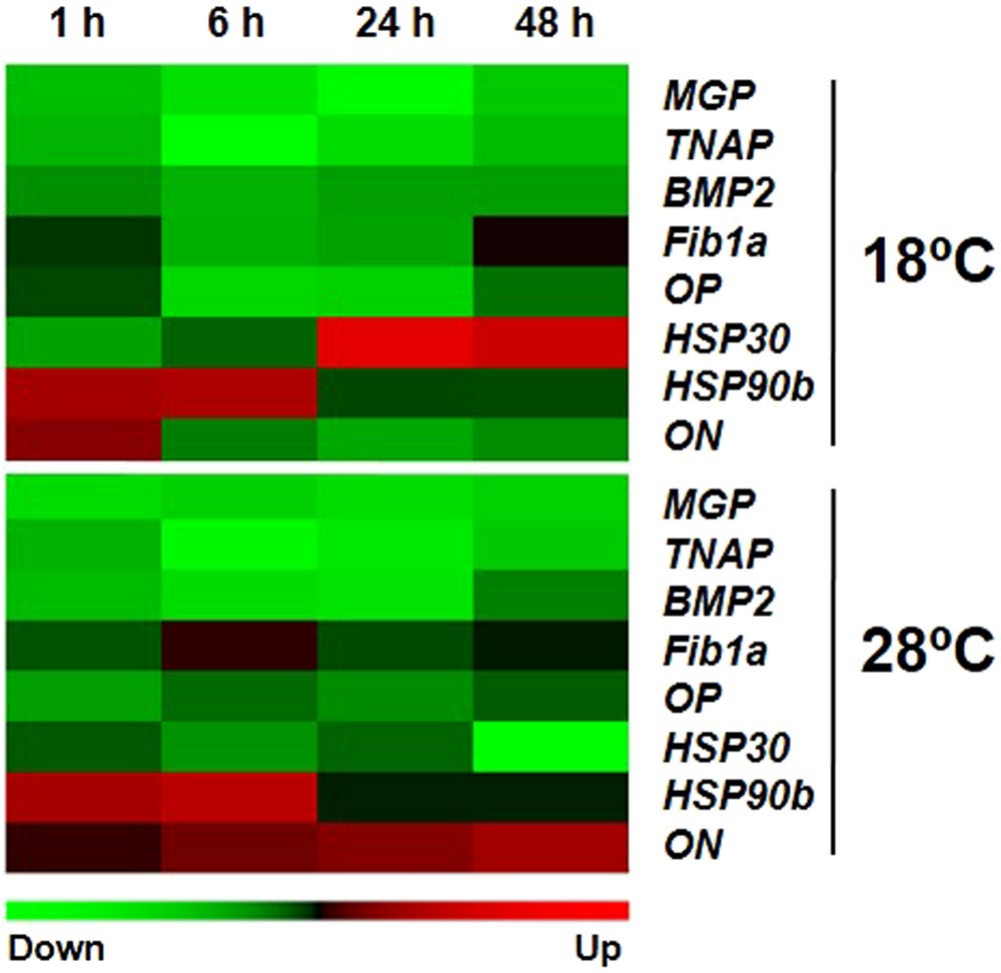
Heat map representing changes in osteogenic genes expression in gilthead sea bream bone-derived primary cell cultures incubated at different temperatures. Quantitative gene expression was relative to the geometric mean of *RPL27* and *Ub* for *MGP*, *TNAP*, *BMP2*, *Fib1a*, *OP*, *HSP30*, *HSP90b* and *ON*. Changes in gene expression were determined in cells after 13 days growing in osteogenic medium (OM) and incubated at two different temperatures (18 and 28 °C) and normalized in relation to the control temperature of 23 °C at 1, 6, 24 and 48 h (n = 5–8). Rows (mRNA transcripts) in the heat map were standardized following a standard score normalization. Red and green shading, respectively, indicate the highest and lowest expression levels, as specified in the scale bar at the bottom of the figure. Each block represents the average standard-score normalization for the 5–8 cultures sampled at each time-point.

The expression of osteogenic gene transcripts from cells incubated in OM at 18 and 28 °C in comparison with the control temperature of 23 °C is represented as a heat map in Fig. 2. The relative expression data obtained at each of the three temperatures can be found in Table S1. For most genes, *MGP*, *TNAP*, *BMP2*, *Fib1a* and *OP*, expression was down-regulated in response to an increase or reduction in temperature. However, this is probably not a generalized non-specific response to change in temperature, since the other osteogenic gene analysed (*ON*) showed a contrary pattern. Similarly, heat shock protein 90b (*HSP90b*) gene expression increased in the first 6 h after temperature challenge and then subsequently decreased. In contrast, mRNA levels of *HSP30* and *ON* responded differently to temperatures. At 18 °C the *HSP30* mRNA levels increased over time, while at 28 °C they fell; the gene expression of *ON* was opposite to *HSP30*, since at 18 °C mRNA levels decreased over time and at 28 °C they increased (Fig. 2).

### Bone development and temperature effects *in vivo*

**Figure 3:**
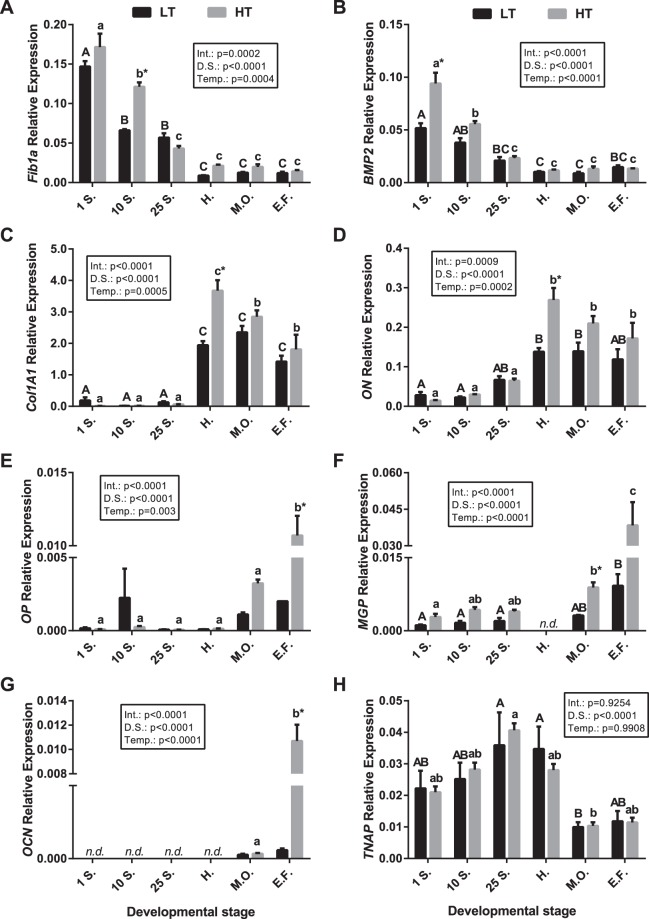
Expression of osteogenic genes in gilthead sea bream embryos and larvae reared at two different temperatures. Quantitative expression relative to *RPS18* of (**A**) *Fib1a*, (**B**) *BMP2*, (**C**) *Col1A1*, (**D**) *ON*, (**E**) *OP*, (**F**) *MGP*, (**G**) *OCN* and (**H**) *TNAP* at different developmental stages (1 somite [1S.], n = 6; 10 somites [10 S.], n = 6; 25 somites [25S.], n = 6; hatch [H.], n = 6; mouth opening [M.O.], n = 6; early flexion [E.F.], n = 2) in fish reared at two temperatures (18 [LT] and 22 °C [HT]). Results are shown as the mean ± s.e.m. Different letters indicate significant differences through time within groups (upper case for LT and lower case for HT) and asterisks indicate significant differences between groups at each developmental stage (p < 0.05). Although it should be taken into account that only 2 samples (each corresponding to a pool of larvae) were available for the developmental stage of early flexion. n.d.: Non-detected, Int.: Interaction, D.S.: Developmental stage, Temp.: Temperature.

Since cell differentiation and proliferation is not always proportional to bone/cartilage formation by osteoblasts/chondrocytes or resorption by osteoclasts, the most suitable predictors of bone mineralization available are: (i) the abundance of ECM proteins, (ii) the activity of cell-type specific enzymes, or (iii) the expression levels of genes encoding these proteins. The expression of key osteogenic genes during embryonic and larval development of gilthead sea bream exposed to two different rearing temperatures was studied and is presented in Fig. 3.

A significant interaction between developmental stage and rearing temperature was observed for all genes studied except *TNAP*. *Fib1a* and *BMP2* had their highest expression during somitogenesis at both temperatures, although expression was significantly higher in the high temperature group (HT, 22 °C) compared to the low temperature group (LT, 18 °C), at 1 somite stage for *BMP2* and at 10 somites stage for *Fib1a* (Fig. 3A,B). The transcription of *Fib1a* and *BMP2* at both temperatures decreased progressively until hatching, after which they remained stable. In contrast, *Col1A1* and *ON* transcript abundance was low during somitogenesis and increased from hatching up to the early flexion stage in both HT and LT groups (Fig. 3C,D). Furthermore, although in the latter stages the mRNA levels of these genes were always higher in the HT group, statistically significant differences were only detected at hatch. The *OP*, *MGP* and *OCN* mRNA levels in the LT and HT groups were very low up until hatch but at mouth opening (*MGP*) and early flexion (*OP* and *OCN*) a significant increase in transcript abundance existed between the two groups, although it should be noted that only 2 samples (pools of larvae) were available for analysis for the latter developmental stage (Fig. 3E–G). Finally, *TNAP* transcripts were very abundant up until hatch and then were significantly down-regulated after mouth opening in both LT and HT groups, and were not significantly different between the temperature groups (Fig. 3H).

**Figure 4:**
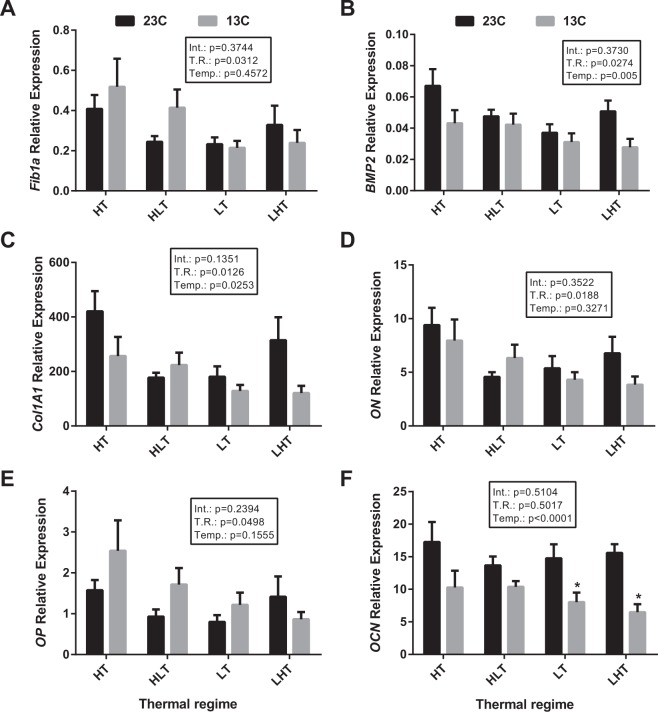
Expression of osteogenic genes in gilthead sea bream juveniles after a temperature challenge *in vivo*. Quantitative expression relative to *RPL27* of (**A**) *Fib1a*, (**B**) *BMP2*, (**C**) *Col1A1*, (**D**) *ON*, (**E**) *OP* and (**F**) *OCN*. Quantitative PCR was performed with samples from gilthead sea bream reared at different temperature regimes during embryogenesis (LT [18–18 °C]; LHT [18–22 °C]; HT [22–22 °C]; HLT [22–18 °C]) and then maintained at 23 °C (LT n = 9, LHT n = 10, HT n = 9, HLT n = 9) or challenged with a drop in water temperature from 23 °C to 13 °C (LT n = 7, LHT n = 9, HT n = 9, HLT n = 9) at 7 months’ post-hatch. Results are shown as the mean ± s.e.m. No significant differences were observed among thermal regimes within each temperature treatment (23 or 13 °C), but asterisks indicate significant differences between temperature treatments for each thermal regime (p < 0.05). Int.: Interaction, T.R.: Thermal regime, Temp.: Temperature. Variation in sample number for different experimental groups was due to the low yield and quality of RNA extracted from some samples.

The analysis of gene expression in bone from 7-month old gilthead sea bream exposed to the four thermal regimes (LT, HT, low-to-high temperature LHT or high-to-low temperature HLT) was not significantly different between the treatment groups (Fig. 4). However, when fish were exposed to a shift in water temperature (23 to 13 °C) a significant reduction in expression was observed for *OCN* in the LT and LHT groups (Fig. 4F).

## Discussion

In the present study we aimed to characterize the molecules involved in osteogenesis in gilthead sea bream and to determine how temperature modified their expression using both *in vitro* and *in vivo* approaches. Moreover, we also investigated the temperature-induced gene responses in bone tissue by challenging juvenile fish that had undergone thermal imprinting during their embryonic and larval stages.

**Figure 5:**
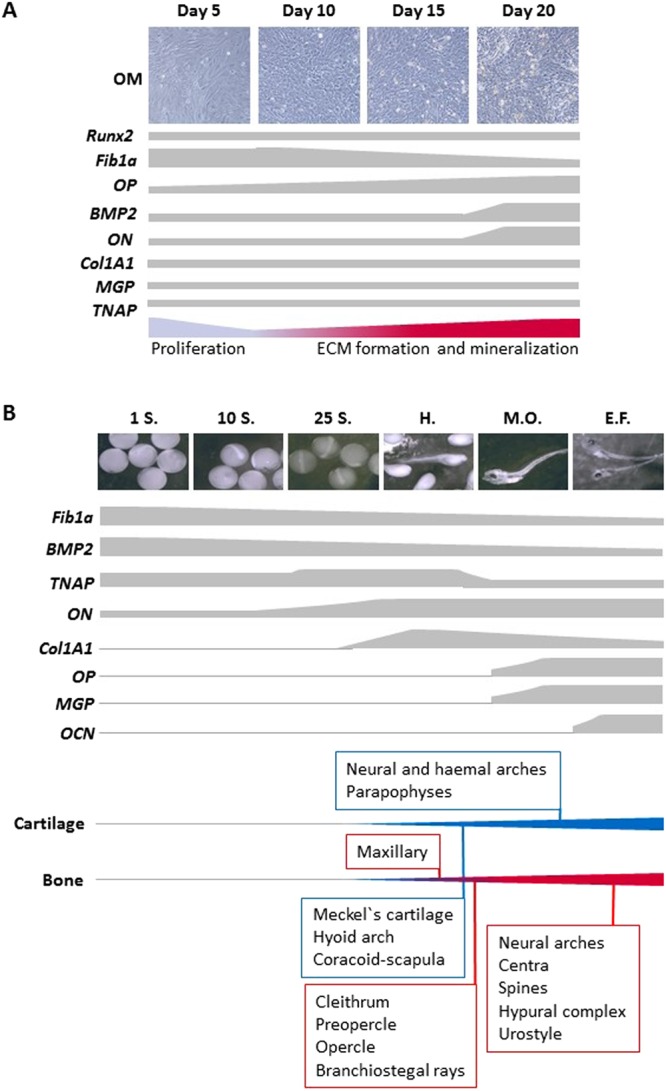
Schematic representation summarizing the expression of osteogenic genes in gilthead sea bream *in vitro* and *in vivo* assays. Representative images of (**A**) bone-derived primary cell cultures growing in osteogenic medium (OM) at days 5, 10, 15 and 20, and (**B**) gilthead sea bream at different developmental stages during embryogenesis (1 somite [1S.]; 10 somites [10S.]; 25 somites [25S.]; hatch [H.]; mouth opening [M.O.]; early flexion [E.F.]). In (**A**), the processes of proliferation and extracellular matrix (ECM) mineralization according to Capilla *et al*.^8^ are schematically indicated with the thick to narrow transition or vice versa representing the approximate rate during culture development. In (**B)**, the developmental osteology of gilthead sea bream indicating the main skeletal structures that are formed is shown^1–3^. Cartilage is represented by the blue line and endochondral and dermal bone formation is represented in red; the blue - red transition represents the onset of endochondral (cartilage replacement bone) formation. The narrow to thick transition of the line for cartilage or bone formation represent the increasing number of structures that develop during development. In sea bream, skeletogenesis begins soon after hatching with the development of cartilaginous and bony structures necessary for exogenous feeding, vision, opening and closing the mouth, expanding and narrowing the oral and branchial areas and propulsion. The comparison between the *in vitro* and *in vivo* results shows that of the genes analysed *Fib1a* is most important during the early development of bone, which is in agreement with its more structural role, while *ON*, *OP*, *MGP* and *OCN* are more abundant in later stages when the deposition of mineral needs to be tightly regulated for the proper formation of bone. In contrast, *BMP2*, *Col1A1* and *TNAP* had divergent expression patterns between the *in vitro* and *in vivo* situation probably due to their diverse roles in tissues during development.

Differentiation of gilthead sea bream bone-derived MSCs into osteoblasts *in vitro* requires the addition of an OM. Shortly after induction, the cells started to change their morphology from a fibroblast-like form to a geometrical shape, and mineralizing nodules became progressively more evident^8^, indicating the characteristic stages of lineage commitment, ECM production and ECM mineralization. These results are similar to those reported by Fernández *et al*.^30^, using another fish bone-derived cell line. These processes are illustrated in the present study by cell morphology and at a transcriptional level (Fig. 5A). Lineage determination and differentiation of osteoblasts from MSCs involves multiple regulatory actors, specific transcription factors, environmental conditions and mineral availability. Among the transcription factors, Runx2 is crucial and it is well-known that it is required for osteoblast differentiation in mammals^31^ and coordinates the expression of genes such as *OP*, *OCN* and alkaline phosphatase^32,33^. In fish, Runx2 is also an important early indicator of the osteogenic capacity of cells^15^ and several authors have shown in fish and mammals that *Runx2* expression increases at very early stages^34–37^. However, in the present study we did not observe significant changes in *Runx2* expression in the cell cultures, which may be because sampling only occurred at day 5. In fact, regulation of *Runx2* levels in gilthead sea bream MSCs undergoing differentiation into osteoblasts was recently detected 6 h after induction (Riera-Heredia, unpublished data). The secreted factor BMP2 is involved in bone commitment and is a widely used growth factor for *in vitro* bone induction^38,39^, although lineage determinant effects of BMPs on MSCs are highly dependent on receptor type and dose^40^. Contrary to expectations, increased *BMP2* expression was not observed in early bone cell cultures. Nonetheless, BMP2 has also been associated later in the process of ECM mineralization and bone nodule formation during MSCs differentiation in mammals^41,42^, which is coherent with the *BMP2* mRNA expression profile detected in the gilthead sea bream MSC cultures described herein. Furthermore, a similar expression pattern was reported in a gilthead sea bream chondrocyte-like VSa13 cell line, since *BMP2* gene expression was also strongly induced during mineralization, but did not occur in the osteoblast-like VSa16 cell line^43^.

Concerning ECM structural elements, Fib1a is produced by osteoblasts and accumulates in the ECM; its expression is higher during the early stages of osteoblast differentiation and declines during cell maturation^44–47^. In our MSC cultures, *Fib1a* expression was highest at day 5 and progressively decreased thereafter, confirming a likely role in the first stages of osteogenesis and cell adhesion^46–48^. Col1A is a component that comprises 90% of the bone ECM, it is expressed during osteoblast differentiation together with some non-collagenous proteins (i.e. ON and OP) and serves as a useful marker of early mineralization. ON acts as a modulator of cell–matrix interactions and has been recognized as a mediator of the early phase of ECM production^49^ and OP is involved in ECM mineralization by regulating calcium phosphate deposition and in bone remodelling by mediating osteoclast attachment to the mineralized ECM during resorption^50–53^. Notwithstanding this timely role, the expression of *ON* and *OP* usually increases during maturation^15,54^, as occurred in our cells, which presented a significant up-regulation by day 20, confirming that these molecules seem to be required for bone mineralization in fish. The results for *OP* agreed with those reported in control *versus* mineralized VSa13 and VSa16 fish cell lines^55^, although under the same conditions *ON* was also shown to be down-regulated by the same authors. During this initial phase of ECM production, an increase in *MGP* and *TNAP* concomitant with *Col1A1*, was expected based on the homology between fish and mammalian systems^56,57^; nevertheless, higher *MGP* mRNA levels were only found at day 20 in OM cells relative to cells growing in GM in our study, but no changes in *TNAP* expression were found. This may indicate that cells during this period were more prone to mineralize than to differentiate as suggested by the *MGP* and *TNAP* mRNA levels; only at later stages of differentiation, has mineralization been found to be negatively regulated by increased *MGP* expression^6^.

Summarizing the *in vitro* characterization analysis, Fib1a appears to be involved in the regulation of the very early stages of the bone cells in culture, possibly in the establishment of the osteoid, while at later stages BMP2, ON, OP and probably MGP, appear to play a role in the consolidation of osteoblast maturation and mineralization of the ECM. Interestingly, the gene expression profiles reported in the present study during embryonic and larval development (Fig. 5B) are concordant with those obtained in our *in vitro* culture model, with the exception of BMP2, demonstrating the relevance and robustness of this *in vitro* system for studies of osteogenesis in this fish species. Furthermore, the gene expression profiles reported herein for *BMP2*, *ON*, *OP* and *OCN* during embryogenesis also agree with those previously presented for this fish species^43,55,58–60^.

*Fib1a* had the highest levels of expression during the somite stages, whereas *Col1A1* and *ON* transcript levels were increased at hatch, confirming their important role in structuring the bone ECM^46–48^ and establishing ECM production^49,54^, respectively. Such up-regulation at hatch could be related *in vivo* with the early formation of the cranial skeleton (neurocranium and spachnocranium) and the caudal fin elements that are of fundamental importance for the proper development of sensory systems, exogenous feeding and survival^61^, since at this stage the vertebral column and fins are not yet differentiated^1,3^. Then, the onset of bone mineralization at mouth opening was marked by increased transcript levels of *MGP* and *OP*, supporting their central role in osteoblast maturation and ECM mineral deposition, mainly in the already formed viscerocranial structures. Finally, *OCN* was significantly up-regulated at early notochord flexion *in vivo* in agreement with its major osteogenic role, although due to the low sample number (n = 2) available in the present study for this stage, further confirmatory analysis is required. The up-regulation of *OCN* could be associated with the appearance at this developmental stage of skeletal structures formed by intramembranous ossification (i.e. vertebral centra, neural and haemal spines) and the mineralization of cartilage replacement bones (i.e. hypurals, neural and haemal arches) since at this time the notochord is still not segmented^1–3^. In fact, although we used whole larvae for gene expression analysis and OCN is not exclusively present in skeletal tissues^31^, it is the most abundant non-collagenous protein in the bone ECM and is considered a late marker of osteoblast differentiation, essential for appropriate maturation of hydroxyapatite crystals and bone mineralization^62–64^. Probably this is why this molecule was undetected in our *in vitro* cultures, because the level of mineralization/maturation of the cells was too low to induce its expression.

Temperature is a primary factor affecting fish larval development, and rearing fish at temperatures outside their optimal range, which in the case of temperate Sparids is below 15 °C and above 22 °C, leads to increased skeletal anomalies^65^. Specifically, for gilthead sea bream, although the usual hatchery temperatures are between 16 and 18 °C^66^, rearing at 22 °C for the entire larval period has been proposed since larvae have less malformations and also have enhanced growth^67^. Overall, while the mechanisms controlling osteogenesis in fish are not fully clarified, it is generally accepted that temperature changes transcription in bone-forming and cartilage-forming cells.

When the responsiveness of gilthead sea bream bone-derived MSCs to a change in temperature was evaluated, an increase in *HSP30* and *HSP90b* transcripts was observed, which may be associated with exposure to stressful conditions as previously reported^68–70^; thus confirming the potentially compromised status of these cells. Most of the osteogenic genes studied in the bone-derived MSCs were down-regulated in response to either an increase or decrease in temperature. A reduction in the osteogenic genes *Col1A1*, *ON* and *OCN* has previously been reported in rat osteoblast primary cultures exposed to hypothermia^71^, and also when Atlantic salmon primary muscle satellite cells that were induced to differentiate into osteoblasts *in vitro* were exposed to a sudden rise in temperature^7^. Thus, although a change in temperature (both an increase and decrease) caused an apparent generalized reduction in gene transcription in our cells, the response was gene specific, as has previously been reported^7,71^. In this sense, our results showed that *ON* was primarily up-regulated in response to temperature change. ON is an ECM glycoprotein that has also been described as a heat shock protein having chaperone-like properties^72,73^. In this context, we cannot eliminate the possibility that the rapid response of *ON* to temperature may be an initial cellular response to stress that is linked with its role in the prevention of collagen denaturation, as has previously been suggested^74^. Overall, the *in vitro* results suggest *ON* gene expression in fish bone may be a candidate indicator of stressful conditions, while the decrease in expression of the other genes may be indicative of a temperature induced check in the osteogenic process.

To evaluate the effects of temperature *in vivo*, fish were reared under two different thermal regimes, and lower expression of all osteogenic genes studied in the embryo and larvae incubated at 18 °C (LT) compared to fish kept at 22 °C (HT) was encountered. Interestingly, the gene expression patterns throughout development were not modified due to incubation temperature, probably since both thermal regimes were within the optimal temperature range for gilthead sea bream rearing^65^. In this sense, although bone development might be accelerated in the HT fish, the consequences with regard to the incidence of malformations is not known. Moreover, in all cases the differences in transcript levels were most significant at the developmental time at which the corresponding gene had its peak of expression: at the stages of 1 and 10 somites for *BMP2* and *Fib1a*, respectively, at hatching for *Col1A1* and *ON* and at early flexion for *OP* and *OCN*. In the latter case substantial differences in gene transcript expression were detected, but since only 2 samples (each corresponding to a pool of larvae) were available for this developmental stage, further confirmation is required. Since the potential temperature effects on fish from different experimental treatments were corrected by sampling using developmental stage as a reference, these data indicate that the most temperature sensitive developmental period is embryogenesis, when imprinting mechanisms can influence the expression of osteogenic genes (i.e. epigenetic modification of chromatin and histones, DNA methylation)^75^. Furthermore, the results of our study indicate that, in common with skeletal muscle and other physiological systems, the skeleton of gilthead sea bream is affected by a short-term change in temperature during embryogenesis. Moreover, in various teleost species the embryonic rearing temperatures produced persistent effects by affecting gene expression in adults even when grown at a common temperature from hatch onwards^76–78^.

Mateus *et al*.^29^, recently demonstrated in gilthead sea bream that the temperature regime applied during early development caused thermal imprinting with long-term effects on bone turnover and gene expression. The present study reinforces the previous observations and identifies, using gene markers as a proxy and an *in vitro* culture model, the molecular and cellular effects. In fact, juvenile fish imprinted with the LT and LHT thermal regimes had reduced transcription of some genes in the bone when the water temperature fell from 23 to 13 °C, suggesting bone formation and mineralization was checked and this may be another negative consequence of winter syndrome. The significant differences observed for *OCN* agree with that reported previously^29^, where the cold challenge in these fish caused a reduction in the bone calcium content and the relative abundance of *OCN* and osteoglycin, a small leucine-rich proteoglycan found in the ECM of bone. In the same study^29^, the LHT and LT groups also had the most notable down-regulation of the endocrine factors controlling bone growth (i.e. insulin-like growth factor 1 and glucocorticoid and thyroid receptors). The reduced expression of some key regulatory and osteogenic factors was proposed to be indicative of impaired responsiveness of bone, suppressing osteoblast differentiation and affecting bone homeostasis and remodelling^79,80^. In support of this notion, hyperthermic Atlantic salmon showed down-regulation of ECM genes (i.e. *Col1A1*, *ON* and *OCN*) and at the juvenile stage had shorter and less mineralized vertebral bodies and a higher rate of skeletal malformations than fish reared under a normal temperature regime^19^. On the other hand, mRNA levels of all the genes under the HT and HLT treatments remained similar irrespective of the rearing temperature of the juveniles. This may show the possible beneficial effects for the skeletal tissue in juveniles and adults, of thermal imprinting during embryogenesis. We hypothesize that under the present experimental conditions, epigenetic mechanisms such as modifications in chromatin and histones or DNA methylation were in place to maintain the expression of the osteogenic genes that had a higher transcript abundance in the HT and HLT groups during their early development, whereas the changes caused by temperature imprinting in the LT and LHT groups were less beneficial. In view of the variable response of the gene transcript expression to a drop in temperature in the different thermally imprinted groups, we conclude that the response is specific and not generalized to either, i) reduced water temperature or ii) the reduced food intake in the 13 °C group (1% *versus* 3% of the controls at 23 °C). The specificity of the response of the thermally imprinted fish to a temperature drop was further supported by the similar level of abundance of the reference genes (also in the *in vitro* study), in the control and challenged fish, and this counters the idea that the results obtained arose from a global repression in transcription due to temperature/nutrient reduction.

Overall, the present study provides novel and detailed data characterizing the gene networks regulating the process of osteogenesis in developing gilthead sea bream exposed to thermal imprinting. We further demonstrated the potential of the gilthead seabream MSCs *in vitro* system as a reliable model to investigate osteogenesis and revealed that it mirrors accurately the data obtained with *in vivo* models. Thus, this study provides, not only better knowledge with regards to bone development and temperature induced effects, but also presents a promising tool for future studies aimed at unravelling the mechanisms underlying the high incidence of skeletal deformities in aquaculture.

## Methods

### *In vitro* studies

#### Primary cultures of bone-derived cells from gilthead sea bream

Gilthead sea bream were obtained from a fish farm in the North of Spain (Tinamenor S.L., Pesués) and maintained in the animal facilities of the Faculty of Biology at the University of Barcelona. Fish were kept in 200 L fiberglass tanks under a 12 h light/12 h dark photoperiod and fed *ad libitum* twice daily with a commercial diet (OptiBream™, Skretting, Burgos, Spain). All animal handling procedures were carried out in accordance with the guidelines of the European Union Council (86/609/EU) and were approved by the Ethics and Animal Care Committee of the University of Barcelona (permit numbers CEEA 243/12 and DAAM 6759). Primary cultures of gilthead sea bream bone-derived MSCs were performed as previously described^8^. Briefly, six fish (average weight 23 g) per culture were used, they were sacrificed by a blow to the head, and the vertebral column was removed and cleaned. The vertebrae were diced-up into smaller fragments using a scalpel and then, two subsequent digestions of 30 and 90 min, respectively, were performed with 0.125% Type II collagenase. Next, the tissue fragments obtained were washed with Dulbecco’s Modified Eagle Medium (DMEM) supplemented with a 1% antibiotic/antimycotic solution (A/A) and plated with complete GM composed of DMEM with 10% fetal bovine serum, 1% Fungizone and 1% A/A and incubated at 23 °C and 2.5% CO_2_. After 1 week, the vertebrae fragments were removed, and the cells collected with 0.25% trypsin-EDTA and routinely sub-cultured for a maximum of 10 passages. All plastic ware was obtained from Nunc (Barcelona, Spain). The trypsin–EDTA and Fungizone solutions were from Invitrogen (El Prat de Llobregat, Spain), and all the other reagents were purchased from Sigma-Aldrich (Tres Cantos, Spain).

#### Characterization of osteogenesis *in vitro*

In order to characterize the stages of development of the cell cultures, trypsinised cells in suspension were counted and plated in 6-well plates with GM at a density of 10^5^ cells per well (n = 8 independent experiments). On the following day (day 0), the media was changed to grow the cells under control (GM) or mineralizing conditions using an OM, which consisted of GM supplemented with 50 µg/ml L-ascorbic acid, 10 mM β-glycerophosphate and 4 mM CaCl_2_. These cell cultures (GM and OM) were then sampled at different time points (days 5, 10, 15 and 20) with pools of 2 wells collected into 1 ml of Tri Reagent® solution (Life Technologies, Alcobendas, Spain) and stored at −80 °C until gene expression analyses were performed.

#### Differentiating bone cells at three temperatures

Cultivated bone-derived cells were grown in OM until day 13 to have differentiated cells in a partly mineralized ECM (n = 7 independent experiments). Then, the cell plates were divided into three different temperature groups (time 0). One group was kept at 23 °C (control), a second group was transferred to an incubator at 18 °C and a third group was moved to 28 °C. Cell samples from the three temperature groups were thereafter harvested for gene expression analyses at the following time points: 1, 6, 24 and 48 h after the temperature challenge. Results from cells incubated at the two experimental temperatures (18 and 28 °C) were analysed relative to cells maintained at the control temperature of 23 °C and standardized (i.e. standard score normalization). A heat map with these values was then generated using PermutMatrix version 1.9.3^81^.

### *In vivo* studies

#### Animals and ethics statement

Rearing of gilthead sea bream embryos, larvae and juveniles was performed at the Institute for Aquaculture and Food Technology Research, (IRTA, Sant Carles de la Ràpita, Spain), in a temperature-controlled seawater recirculation system (IRTAmar™). Animal handling procedures were carried out in accordance with the guidelines of the European Union Council (86/609/EU) and were approved by the Ethics and Animal Care Committee of IRTA (4998-T9900002).

#### Embryogenesis characterization at two temperatures

The effect of temperature on embryonic and larval development was determined by rearing fish under a constant temperature of 18 °C (LT) or 22 °C (HT). Fertilized eggs (110 mL/tank) of gilthead sea bream (fertilization rate of 92%) were maintained in duplicate 30 L tanks from embryogenesis up until the larval-juvenile transition. A detailed description of the experiment is provided in García de la serrana *et al*.^75^. Samples of each stage, 6 per tank, were removed by quickly netting the larvae, sacrificed with an overdose of anaesthetic (150 mg/L of MS-222; Sigma, Tres Cantos, Spain) and snap frozen in liquid nitrogen or fixed in 4% paraformaldehyde. Embryos and larvae were sampled at: 1 (n = 6), 10 (n = 6) and 25 somites (S., n = 6), hatch (H., n = 6), mouth opening (M.O., n = 6) and early notochord flexion (E.F., n = 2), and to compensate for the potential effects of temperature on the developmental progression, the samples of fish from the two different experimental groups were collected using developmental stage as the reference.

#### Temperature drop challenge

For this experiment, fish reared under four different thermal regimes during embryonic and larval development were studied. A schematic representation of the experimental trial and sampling details can be found in Mateus *et al*.^29^, where biometric and bone homeostasis parameters are reported. After hatching, the temperature was either maintained (LT, 18 °C or HT, 22 °C groups, respectively) or changed from 22 to 18 °C (HLT) or 18 to 22 °C (LHT) up until the larval-juvenile transition. Juvenile fish were then transferred to duplicate 2000 L tanks per treatment connected to a recirculating sea water system (5–10% water renewal per day, IRTAmar™) at 22–23 °C and were fed 3% body mass (w/w) with a commercial diet (OptiBream™, Skretting, Spain).

For the cold challenge experiment, fish were age matched (7 months’ post-hatch), although significant differences (P < 0.001) in weight and length existed between the fish reared under the different thermal regimes during embryogenesis as previously reported^29^. Duplicate tanks of fish from the four different thermal regimes, were either, (a) maintained at the same temperature of 23 ± 1 °C or (b) exposed to a temperature challenge of 13 ± 1 °C for 15 days. The circuit consisted of 200 L fibreglass tanks in a semi-closed sea water system at pH 7.5–8.0, 35–36‰ salinity and >80% oxygen saturation and maintained under a 12 h light/12 h dark photoperiod. Fish from the control group were fed at the rate of 3% body weight daily using a commercial diet (OptiBream™), while fish from the cold challenge were fed at a rate of only 1%, and uneaten food was siphoned daily from the bottom of the experimental tanks. These feeding ratios were adjusted to the differences observed in intake due to rearing temperatures during acclimation to keep the tanks clean and with good water quality conditions. Ten fish from each condition were sacrificed with an overdose of phenoxyethanol (450 ppm) and samples of vertebrae were collected. No mortality occurred during the experimental trial and the gilthead sea bream exhibited no signs of distress during the experiment.

### Gene expression analyses

#### RNA extraction and cDNA synthesis

Total RNA was isolated using Tri Reagent® following the manufacturer’s instructions and quantified using a NanoDrop2000 spectrophotometer (Thermo Scientific, Alcobendas, Spain), and the quality analysed by 1% (w/v) agarose gel electrophoresis. One µg of total RNA per sample was DNase treated (Life Technologies, Alcobendas, Spain) and used to synthesise first-strand cDNA using the Transcriptor First Strand cDNA Synthesis Kit (Roche, Sant Cugat del Vallès, Spain), following the manufacturers’ instructions.

#### Quantitative real-time PCR (qPCR)

The qPCR assays were conducted according to the requirements of the MIQE guidelines^82^ using a CFX384™ Real-Time System (Bio-Rad, El Prat de Llobregat, Spain). Prior to the analyses, the specificity of the reaction, absence of primer-dimers, as well as the most appropriate cDNA working dilution for each assay was determined by running a dilution curve with a pool of samples. Reactions were performed in triplicate (methodological replicates) and contained cDNA, iQ SYBR Green Supermix (Bio-Rad) and 250 nM (final concentration) of sense and antisense primers (Table 1). The protocol consisted of 1 cycle of 3 min at 95 °C and 40 cycles of 10 s at 95 °C and 30 s at 55–68 °C (primer dependent, see Table 1), followed by an amplicon dissociation analysis from 55 to 95 °C with a 0.5 °C increase every 30 s. A single peak was observed for each of the qPCR reactions and this confirmed reaction specificity. SYBR Green fluorescence was recorded during the annealing-extending phase of cycling. Target transcript abundance was analysed using the delta-delta Ct method^83^ with the CFX Manager Software (Bio-Rad). In the *in vitro* studies, gene expression results were normalized using the geometric mean of ribosomal protein S18 (*RPS18*) and elongation factor 1 alpha (*EF1a*) for the culture development experiment and ribosomal protein L27 (*RPL27*) and ubiquitin (*Ub*) for the temperature experiment. *RPS18* and *RPL27* were used to standardise the gene expression during the *in vivo* experiments of embryogenesis and the adult temperature challenge, respectively. In each case, the reference gene(s) selected were those for which stability was confirmed by running the GeNorm algorithm implemented in the CFX Manager Software (Bio Rad).

**Table 1:** Primers used for real-time quantitative PCR.

Gene	Primer sequence (5′-3′)	Tm (°C)	Acc. Num.	Efficiency (%)
*BMP2*	F: GGAGAAGCAGCGTGGATTAAACACGAAT	68	AY500244	103.9
	R: GGCCTGCGCCTCAGTCCAAACATATT			
*Col1A1*	F: GAGATGGCGGTGATGTGGCGGAGTC	68	DQ324363	91.7
	R: GCCTGGTTTGGCTGGATGAAGAGGG			
*EF1α*	F: CTTCAACGCTCAGGTCATCAT	60	AF184170	94.2
	R: GCACAGCGAAACGACCAAGGGGA			
*Fib1a*	F: CGGTAATAACTACAGAATCGGTGAG	60	FG262933	101.8
	R: CGCATTTGAACTCGCCCTTG			
*HSP30*	F: GGTGACTGACGGGAAAGAGA	60	GU060312	93.1
	R: CTGAGGAGGAGGTGCTGTTC			
*HSP90b*	F: TTCACGCATGGAAGAAGTTG	56	DQ012949	86.0
	R: GGTCCACCACACAACATGAA			
*MGP*	F: TGTGTAATTTATGTAGTTGTTCTGTGGCATCTCC	68	AY065652	89.2
	R: CGGGCGGATAGTGTGAAAAATGGTTAGTG			
*OCN*	F: TCCGCAGTGGTGAGACAGAAG	60	AF048703	90.7
	R: CGGTCCGTAGTAGGCCGTGTAG			
*ON*	F: AGGAGGAGGTCATCGTGGAAGAGCC	68	AY239014	97.1
	R: GTGGTGGTTCAGGCAGGGATTCTCA			
*OP*	F: AAAACCCAGGAGATAAACTCAAGACAACCCA	68	AY651247	95.3
	R: AGAACCGTGGCAAAGAGCAGAACGAA			
*RPL27*	F: AAGAGGAACACAACTCACTGCCCCAC	68	AY188520	97.4
	R: GCTTGCCTTTGCCCAGAACTTTGTAG			
*RPS18*	F: AGGGTGTTGGCAGACGTTAC	60	AM490061	100.3
	R: CTTCTGCCTGTTGAGGAACC			
*Runx2*	F: ACCCGTCCTACCTGAGTCC	60	JX232063	96.1
	R: AGAAGAACCTGGCAATCGTC			
*TNAP*	F: CATCGCAACCCTTTTCACAGTCACCCG	68	AY266359	103.2
	R: AACAGTGCCCAAACAGTGGTCCCATTAGC			
*Ub*	F: CGGAAGTAAGAGGAACCAACAC	56	AM955423	78.4
	R: AAGCAGTCAGAATGCAAAGTCA			

F, forward primer; R, reverse primer; Tm, annealing temperature; Acc. Num., accession number.

### Statistical analyses

Statistical analyses of all parameters were performed in SPSS Statistics version 20 (IBM, Armonk, NY, USA). Normality was analysed using the Shapiro-Wilk test and homogeneity of variance using a Levene’s test. Statistical significance was assessed by two-way analysis of variance (two-way ANOVA) followed by Tukey *post-hoc* test. Significant differences were taken at p < 0.05 for all statistical tests performed. Data are presented as mean ± standard error of the mean (s.e.m.). Significant differences through time within groups (i.e. days in culture, developmental stages or thermal regimes) are shown with different letters and significant differences between treatments (i.e. culture media or rearing temperature) are shown with an asterisk.

### Data availability

All data generated or analysed during this study are included in this published article (and its Supplementary Information files).

## Electronic supplementary material

Table S1
