# Supplementary material for: Temperature responsiveness of gilthead sea bream bone; an in vitro and in vivo approach
Source: Sci Rep. 2018 Jul 25;8:11211. doi: 10.1038/s41598-018-29570-9 (PMC6060158; doi:10.1038/s41598-018-29570-9)
Supplement: Supplementary file 1 — Table S1 [file 41598_2018_29570_MOESM1_ESM.pdf]

Temperature responsiveness of gilthead sea bream bone; an *in vitro* and *in vivo* approach

Natàlia Riera-Heredia[1], Rute Martins[2], Ana Patrícia Mateus[2], Rita A. Costa[2], Enric Gisbert[3], Isabel Navarro[1], Joaquim Gutiérrez[1], Deborah M. Power[2] and Encarnación Capilla[1]*

Table S1. Expression of osteogenic genes in gilthead sea bream bone-derived primary cell cultures *in vitro* at three different temperatures. Quantitative gene expression relative to the geometric mean of *RPS18* and *Ub* for *MGP*, *TNAP*, *BMP2*, *Fib1a*, *OP*, *HSP30*, *HSP90b* and *ON*. Cells were growing in osteogenic medium (OM) for 13 days at 23ºC of temperature and then divided into three temperature groups (18, 23 and 28ºC) and sampled at 1, 6, 24 and 48 h. The results are shown as the mean ± s.e.m. (n=5-8).

		**1 h**	**6 h**	**24 h**	**48 h**
MGP	**18ºC**	1.62±0.80	1.31±0.66	0.70±0.37	1.49±0.76
	**23ºC**	1.37±0.74	1.56±0.66	1.76±0.00	1.49±0.67
	**28ºC**	0.84±0.43	1.17±0.66	1.80±1.27	1.59±1.13
TNAP	**18ºC**	0.72±0.40	0.42±0.23	0.62±0.29	1.06±0.57
	**23ºC**	0.66±0.35	1.21±0.87	0.97±0.00	0.91±0.44
	**28ºC**	0.78±0.53	1.08±0.79	0.65±0.40	0.74±0.39
BMP2	**18ºC**	3.06±0.82	2.23±0.18	2.00±0.47	1.77±0.30
	**23ºC**	3.37±0.95	4.10±1.16	3.01±0.00	2.55±0.57
	**28ºC**	3.37±0.95	4.10±1.16	3.01±0.00	2.55±0.57
Fib1a	**18ºC**	0.94±0.22	0.52±0.12	0.54±0.13	0.79±0.15
	**23ºC**	0.85±0.12	0.81±0.16	0.79±0.00	0.71±0.18
	**28ºC**	0.91±0.23	1.02±0.29	0.80±0.21	0.79±0.18
OP	**18ºC**	0.66±0.17	0.30±0.09	0.48±0.14	1.23±0.51
	**23ºC**	0.59±0.17	0.61±0.16	0.89±0.00	1.04±0.32
	**28ºC**	0.60±0.33	0.70±0.33	0.77±0.26	0.90±0.10
HSP30	**18ºC**	1.57±0.58	2.22±0.84	2.85±1.38	7.07±3.09
	**23ºC**	1.76±0.59	1.81±0.69	1.72±0.00	1.71±0.89
	**28ºC**	1.69±0.36	1.71±0.63	0.56±0.23	0.43±0.13
HSP90b	**18ºC**	1.11±0.07	1.19±0.08	1.04±0.07	0.97±0.13
	**23ºC**	0.66±0.09	0.67±0.21	1.04±0.00	1.04±0.06
	**28ºC**	1.15±0.12	1.36±0.18	1.13±0.08	1.07±0.15
ON	**18ºC**	0.74±0.13	0.45±0.08	0.43±0.08	0.61±0.16
	**23ºC**	0.48±0.11	0.58±0.10	0.69±0.00	0.64±0.13
	**28ºC**	0.63±0.14	0.88±0.27	1.02±0.20	1.15±0.23
